# *Lmo* Mutants Reveal a Novel Role for Circadian Pacemaker Neurons in Cocaine-Induced Behaviors

**DOI:** 10.1371/journal.pbio.0020408

**Published:** 2004-11-23

**Authors:** Linus T.-Y Tsai, Roland J Bainton, Justin Blau, Ulrike Heberlein

**Affiliations:** **1**Department of Anatomy, Program in Neuroscienceand Medical Science Training Program, University of California, San Francisco, CaliforniaUnited States of America; **2**Department of Anesthesia, University of CaliforniaSan Francisco, CaliforniaUnited States of America; **3**Department of Biology, New York UniversityNew York, New YorkUnited States of America; **4**Department of Anatomy, Programs in Neuroscience and Developmental BiologyUniversity of California, San Francisco, CaliforniaUnited States of America

## Abstract

*Drosophila* has been developed recently as a model system to investigate the molecular and neural mechanisms underlying responses to drugs of abuse. Genetic screens for mutants with altered drug-induced behaviors thus provide an unbiased approach to define novel molecules involved in the process. We identified mutations in the *Drosophila* LIM-only (LMO) gene, encoding a regulator of LIM-homeodomain proteins, in a genetic screen for mutants with altered cocaine sensitivity. Reduced *Lmo* function increases behavioral responses to cocaine, while *Lmo* overexpression causes the opposite effect, reduced cocaine responsiveness. Expression of *Lmo* in the principal *Drosophila* circadian pacemaker cells, the PDF-expressing ventral lateral neurons (LN_v_s), is sufficient to confer normal cocaine sensitivity. Consistent with a role for *Lmo* in LN_v_ function, *Lmo* mutants also show defects in circadian rhythms of behavior. However, the role for LN_v_s in modulating cocaine responses is separable from their role as pacemaker neurons: ablation or functional silencing of the LN_v_s reduces cocaine sensitivity, while loss of the principal circadian neurotransmitter PDF has no effect. Together, these results reveal a novel role for *Lmo* in modulating acute cocaine sensitivity and circadian locomotor rhythmicity, and add to growing evidence that these behaviors are regulated by shared molecular mechanisms. The finding that the degree of cocaine responsiveness is controlled by the *Drosophila* pacemaker neurons provides a neuroanatomical basis for this overlap. We propose that *Lmo* controls the responsiveness of LN_v_s to cocaine, which in turn regulate the flies' behavioral sensitivity to the drug.

## Abstract 2

Expression of the *Drosophila* LIM-only (LMO) gene in circadian pacemaker cells is required for the normal behavioral sensitivity of these animals to cocaine

## Introduction

Cocaine, a naturally occurring plant alkaloid, is the prototype addictive psychomotor stimulant. It elicits a variety of acute behavioral changes ranging from mood elevation, disinhibition, and motor activation at low doses to compulsive stereotypies and psychosis at higher doses (Gawin 1991). Long-term cocaine use generally results in tolerance to many of its subjective effects, an increased craving towards the drug, and, eventually, drug abuse and addiction.

Cocaine's primary mechanism of action is to bind and inhibit plasma membrane monoamine transporters, thereby increasing synaptic monoamine neurotransmitter levels and potentiating their actions. Cocaine's direct role in increasing dopamine (DA) in the nucleus accumbens via inhibition of the DA transporter (DAT) has contributed to the prevalent DA hypothesis of drug addiction, which posits that the shared ability of drugs of abuse to increase DA in the nucleus accumbens underlies their reinforcing properties (Wise and Bozarth 1985; Kuhar et al. 1991). More recent animal studies, however, have shown that cocaine still elicits a robust conditioned place preference in mice with genetic deletions of DAT, suggesting that the rewarding properties of cocaine are not solely mediated by its action on DAT (Sora et al. 1998). Additional studies on mice in which the serotonin transporter or norepinephrine transporter has been deleted, together with studies using selective serotonin transporter or norepinephrine transporter inhibitors, have suggested roles for both serotonin and norepinephrine systems in mediating cocaine's rewarding properties (Uhl et al. 2002). These data show that the molecular bases for cocaine's psychostimulant and reinforcing properties are more complicated than once thought, and that a combination of actions at multiple sites may mediate its effects. Indeed, multiple genes and signaling pathways have been implicated in the stimulant and rewarding properties of cocaine in mice (Laakso et al. 2002).

The fruit fly Drosophila melanogaster has been advanced as a useful model system for identifying novel genes that regulate behavioral responses to drugs of abuse including cocaine (Wolf and Heberlein 2003). Several of cocaine's most characteristic properties have been recapitulated in flies. First, cocaine induces motor behaviors in flies (see below) that are remarkably similar to those observed in mammals (McClung and Hirsh 1998; Bainton et al. 2000). Second, repeated cocaine administration induces behavioral sensitization (McClung and Hirsh 1998), a form of behavioral plasticity believed to underlie certain aspects of addiction (Robinson and Berridge 1993; Schenk and Partridge 1997). Finally, a key role for dopaminergic systems in mediating cocaine's effects has been demonstrated through both pharmacologic and genetic methods (Bainton et al. 2000; Li et al. 2000). More importantly, *Drosophila* studies have identified genes and pathways whose role in cocaine responsiveness had not been anticipated (Hirsh 2001; Rothenfluh and Heberlein 2002). For instance, the *Drosophila* circadian gene *period* was identified as a necessary mediator of cocaine sensitization (Andretic et al. 1999). Subsequently, mice carrying various *period* mutations were found to have altered cocaine sensitization and conditioned place preference (Abarca et al. 2002).

In order to identify novel molecules and pathways involved in behavioral responses to cocaine, we carried out a genetic screen for *Drosophila* mutants with altered acute responses to cocaine. Here, we report the phenotypic and molecular characterization of mutations in the *Drosophila* LIM-only gene, *Lmo* (also called *Beadex [Bx]*), isolated due to their increased sensitivity to cocaine-induced loss of negative geotaxis. The products of *Drosophila* and mammalian *Lmo* genes modulate the function of LIM-homeodomain (LIM-HD) proteins (Milan et al. 1998; Retaux and Bachy 2002), which in turn regulate various aspects of nervous system development, including the specification of neural identity (Thor and Thomas 1997; Hobert and Westphal 2000; Shirasaki and Pfaff 2002; Tsalik et al. 2003). We find that cocaine sensitivity is inversely related to the levels of *Lmo* function: reduced function causes increased sensitivity, while increased function causes resistance. This bidirectional regulation of cocaine responsiveness is mediated by *Lmo* function in a small set of neurons, the ventral lateral neurons (LN_v_s), which are the primary circadian pacemaker cells in *Drosophila* (Helfrich-Förster 1997; Renn et al. 1999). Like other mutants that affect LN_v_ function, *Lmo* mutants show altered circadian locomotor behaviors. However, the roles of the LN_v_s in regulating cocaine sensitivity and circadian behavioral rhythms are genetically separable, as mutants lacking the neuropeptide PDF—the only known functional output of these neurons—show normal cocaine sensitivity. Thus, *Lmo* defines a novel role for the circadian pacemaker cells in regulating behavioral responses to cocaine.

## Results

### Loss- and Gain-of-Function Mutations in the *Lmo* Locus Show Altered Cocaine Sensitivity

**Figure 1:**
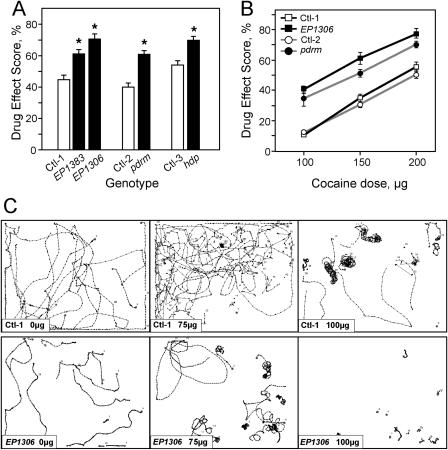
*Lmo* Loss-of-Function Mutants Show Increased Sensitivity to Cocaine (A) Cocaine phenotypes of various *Lmo* mutants. Male flies hemizygous for the indicated *Lmo* alleles (and their appropriate genetic controls) were exposed to 150 μg of cocaine and tested in the crackometer as described in Materials and Methods. Compared to their control (Ctl-1), *EP1383* (*p <* 0.02) and *EP1306* (*p <* 0.001) flies show significantly increased sensitivity to cocaine. Similarly, compared to their respective controls, *pdrm* and *hdp* flies are significantly more sensitive to cocaine (*p <* 0.001). Asterisks denote significant differences from controls (Student's paired *t*-test assuming equal variance); *n* = 20 experiments. (B) Cocaine dose–response. *EP1306* flies (filled squares) and *pdrm* flies (filled circles) and their respective controls were exposed to the indicated doses of cocaine. At each dose, the responses of *EP1306* and *pdrm* flies are significantly higher than their controls (*p <* 0.001, *n* = 16–20 experiments). (C) *EP1306* flies show alterations in cocaine-induced locomotor patterns of activity. Flies were exposed to 0, 75, or 100 μg of cocaine, as indicated, for 1 min. Representative traces shown correspond to 30 s of recorded activity of about ten flies starting 1 min after the end of cocaine exposure (*n* ≥ 4). Top panels show response of control flies to indicated amounts of cocaine; bottom panels show activity of *EP1306* flies after cocaine administration. Ctl-1 is *EP1631,* Ctl-2 is P[GAL4] line *8.142,* and Ctl-3 is *w^1118^*.

To identify novel molecules involved in cocaine-related behaviors, we carried out a genetic screen for mutants with altered acute responses to volatilized freebase cocaine using the crackometer, a simple assay that measures cocaine-induced loss of negative geotaxis (Bainton et al. 2000). Screening of 400 first chromosome P-element insertions from the EP collection (Rørth 1996) led to the identification of five mutants with reduced cocaine sensitivity and seven with increased sensitivity. Two of the mutants conferring increased cocaine sensitivity, *EP1306* and *EP1383* (Figure 1A), carry a P-element insertion in the promoter region of the *Drosophila Lmo* locus (Milan et al. 1998; Zeng et al. 1998). *Drosophila* LMO protein has been shown to inhibit the activity of the LIM-HD transcription factor apterous through its interactions with the LIM-HD activator Chip (Milan and Cohen 1999; Weihe et al. 2001).

Independently, we isolated a P[GAL4] insertion in the *Lmo* locus, dubbed *pipedream (pdrm),* that also showed increased cocaine sensitivity (Figure 1A). Finally, *heldup (hdp)* mutant flies, which carry well-characterized loss-of-function mutations in *Lmo* (Milan et al. 1998), displayed increased sensitivity to cocaine that is similar in magnitude to that of the *EP1306* line (Figure 1A). This increased sensitivity was observed at all cocaine doses tested (Figure 1B and 1C; data not shown). The *EP1306* and *hdp* alleles consistently demonstrated stronger phenotypes than *EP1383* and *pdrm*. All mutants tested performed within the normal range in task-specific baseline behaviors (see Materials and Methods). Finally, we were able to revert the cocaine phenotype of *EP1306* by excision of the P element (data not shown). Taken together, these data demonstrate that loss-of-function mutations in *Lmo* result in increased sensitivity to cocaine.

The dominant *Beadex (Bx)* mutations were first described in 1925 by Morgan, Bridges, and Sturtevant, and named for their characteristic beaded wing margin (Morgan et al. 1925). More recently, *Bx* mutants were shown to be overexpression alleles of *Lmo* (Milan et al. 1998; Shoresh et al. 1998; Zeng et al. 1998). Overexpression results from the insertion of naturally occurring transposable elements into the 3′ UTR of *Lmo,* which leads to transcript stabilization (Shoresh et al. 1998). The resultant overexpression of *Lmo* causes decreased apterous activity in the dorsal compartment of the wing and, consequently, disorganization of the wing margin (Milan and Cohen 1999, 2000).

**Figure 2:**
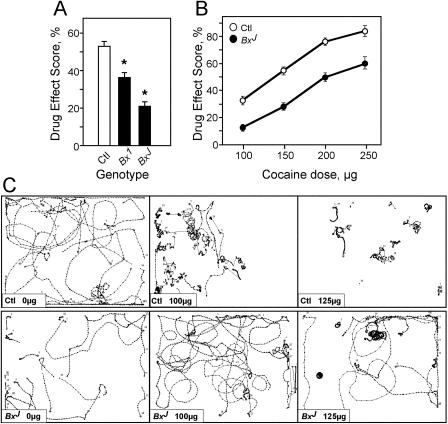
*Lmo* Gain-of-Function *Bx* Alleles Show Reduced Sensitivity to Cocaine (A) Cocaine phenotypes of *Bx* mutants. Male flies hemizygous for *Bx* alleles *Bx^1^* or *Bx^J^* show significant reductions in sensitivity to cocaine compared to control (Ctl) flies (*p <* 0.001, *n* = 12 experiments). Asterisks denote significant differences from control (Student's paired *t*-test assuming equal variance). (B) Dose–response. *Bx^J^* flies (filled circles) show reduced sensitivity compared to Ctl flies (open circles) at all doses tested (*p <* 0.001, *n* = 16–20 for all doses except for 250 μg, where *p* = 0.0015, *n* = 8). Two additional *Bx* alleles (*Bx^2^ and Bx^3^*) had similar phenotypes to *Bx^1^* (not shown). (C) *Bx^J^* flies show alterations in cocaine-induced locomotor patterns of activity. Flies were exposed to 0, 100, or 125 μg of cocaine, as indicated, for 1 min. Representative traces shown are 30 s of recorded activity of about ten flies starting 30 or 60 s after the end of cocaine exposure (*n* ≥ 3). Top panels show response of control flies to indicated amounts of cocaine; bottom panels show activity of *Bx^J^* flies after cocaine administration. Ctl flies are *w^1118^*.

We tested multiple available *Bx* alleles, and found that they all caused resistance to the acute effects of cocaine at every dose tested (Figure 2A and 2B). The strength of the cocaine resistance phenotype correlated well with the severity of the beaded wing-margin phenotype, *Bx^J^* > *Bx^1^* = *Bx^2^* = *Bx^3^* (Figure 2A and 2B; data not shown). Because in *Bx* mutants *Lmo* transcripts are stabilized in their normal spatiotemporal pattern (Shoresh et al. 1998), the observed cocaine phenotypes likely result from overexpression, rather than from misexpression of *Lmo*. Taken together with the loss-of-function effects described above (see Figure 1A and 1B), these data reveal a graded behavioral response to cocaine that is inversely related to *Lmo* levels.

In order to study *Lmo* mutant behavior in more detail, we recorded the cocaine-induced patterns of locomotor activity of control and *Lmo* flies using a movement tracking system (Bainton et al. 2000; Wolf et al. 2002). Upon mock exposure, control flies showed robust locomotor activity, generally walking in straight lines (Figure 1C, top left panel). At relatively low doses of cocaine (75 and 100 μg), flies engaged in stereotyped circling patterns of activity (Figure 1C, top right panels). At higher doses (125–200 μg), flies began to show spasmodic movements (seen as zigzag patterns in movement) and severe hypokinesis (data not shown; Bainton et al. 2000). *EP1306* flies, while showing a reduced baseline speed, showed normal patterns of activity upon mock exposure (Figure 1C, bottom left panel). Upon exposure to cocaine, these mutant flies showed a shift in the dose–response relationship. At low doses (75 μg), *EP1306* flies showed a marked increase in stereotyped circling behavior compared to controls (Figure 1C, bottom middle panel), while at higher doses (100 μg), the mutant flies were more likely to be spasmodic or akinetic (Figure 1C, bottom right panel). Thus, despite the reduced speed observed in the mock exposures, increases in specific cocaine-induced locomotor behaviors demonstrate that *EP1306* flies have a shifted dose–response to cocaine: mutant flies exposed to 75 μg of cocaine behaved similarly to control flies exposed to 100 μg of the drug.

*Bx^J^* flies also showed changes in walking patterns that are consistent with a shift in the cocaine dose–response relationship. After exposure to 100 μg of drug, most control flies showed slow circling behavior and some were akinetic (Figure 2C, top middle panel). *Bx^J^* flies were much less affected, showing increased locomotion (mostly in straight lines), decreased slow circling, and almost no akinesia (Figure 2C, bottom middle panel). At 125 μg, control flies showed very little movement (Figure 2C, top right panel), while most *Bx^J^* flies continued to show circling behaviors seen in control flies at lower doses (Figure 2C, bottom right panel). *Bx^J^* flies, like *EP1306* flies, showed reduced activity upon mock exposure (Figure 2C, bottom left panel). Despite this reduced activity, these two mutants showed very different sensitivities to cocaine: *EP1306* flies were more affected and *Bx^J^* flies were less affected, suggesting that the reduced activity upon mock exposure is unrelated to the cocaine response. Long-term activity recordings revealed no differences between *Bx^J^, EP1306,* and control lines (data not shown; see below).

In summary, using two different behavioral assays we show that flies carrying loss-of-function mutations in *Lmo* display increased sensitivity to the effects of cocaine on locomotor behaviors, while gain-of-function mutations show the converse effect, a reduced response to the drug. This inverse relationship between *Lmo* gene activity and drug responsiveness suggests that *Lmo* may regulate the expression of genes that might play a direct role in controlling cocaine responses (see Discussion).

### Molecular Characterization of *Lmo* Mutants

**Figure 3:**
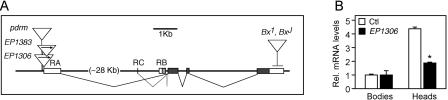
Molecular Structure of the *Lmo* Locus (A) A genomic map of the *Lmo* locus. Three different first exons can be utilized, forming the basis for three alternative transcripts. Exon RA-1 is separated from the alternative start sites RB-1 and RC-1 by a large (∼30 kb) intron. *EP1306, EP1383,* and *pdrm* carry insertions 25, 73, and 91 bp, respectively, upstream of the exon RA-1 transcriptional start site. Arrows within the EP elements refer to the orientation of the insertion and the expected direction of inducible expression via UAS sites contained within the EP element. *Bx* alleles are insertions of natural transposons into the 3′ UTR of the *Lmo* gene that have been shown to stabilize *Lmo* transcript (Shoresh et al. 1998). Protein-coding exons are shaded. (B) Expression of the *Lmo* RA transcript is enriched in *Drosophila* heads, and is reduced in the *EP1306* mutant. RNA was isolated from heads and bodies; after cDNA synthesis, quantitative RT-PCR was performed using primers specific to the RA transcript of *Lmo* in addition to primers to a reference transcript, the ribosomal protein *rp49*. Relative abundance is expressed as fold increase over control *(EP1631)* body mRNA. No detectable amplification was seen in RNase-treated controls (data not shown). Error bars represent standard error of the mean. Asterisk denotes significant difference from control (Student's paired *t*-test assuming equal variance; *p <* 0.001, *n* = 3).

**Figure 4:**
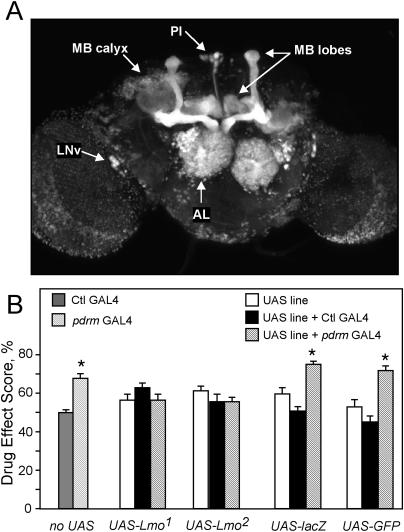
*pdrm*'s GAL4 Expression Is Sufficient to Drive *Lmo*-Transgene-Mediated Rescue of Cocaine Sensitivity (A) *pdrm*'s GAL4 expression pattern. *UAS-GFP* reveals GAL4 expression pattern of the *pdrm* enhancer-trap insertion in MB lobes and calyces, ALs, the large cell bodies of the peptidergic LN_v_s, and neurons of the pars intercerebralis (PI). (B) *Lmo* expression restores wild-type cocaine responses. In the absence of a *UAS* transgene (“no UAS” columns), hemizygous male *pdrm* flies (hatched bar) are more sensitive than controls (Ctl GAL4 is line *8.142,* solid bar), as shown before in Figure 1A. Male flies hemizygous for *pdrm* and heterozygous for either of two *UAS-Lmo* transgenes (*UAS-Lmo^1^* and *UAS-Lmo^2^;* hatched black bars), show normal cocaine sensitivity when compared to either *UAS-Lmo* transgene alone (white bars) or *UAS-Lmo* transgenes in the presence of a control GAL4 line (Ctl GAL4, line *8.142;* black bars). To control for non-specific effects of transgene overexpression, *UAS-GFP* and *UAS-lacZ* transgenes were also driven by *pdrm* GAL4. Male flies were hemizygous for *pdrm* (or heterozygous for the control GAL4 insertion) and heterozygous for the specific *UAS* transgene. One-way ANOVA revealed a significant effect of genotype in the *UAS-lacZ* (*F* = 17.4, *p <* 0.001), *UAS-GFP* (*F* = 19.47, *p <* 0.001), or no *UAS* transgene (*F* = 4.1, *p <* 0.001) groups, but not in either of the *UAS-Lmo* transgene groups (*F* = 1.58, *p* = 0.22 and *F* = 1.21, *p* = 0.31 for *UAS-Lmo^1^* and *UAS-LMO^2^*, respectively); thus, *UAS-Lmo* expression specifically restores normal cocaine sensitivity to *pdrm* flies. Post hoc pairwise planned comparisons, with the critical *p*-value adjusted to 0.025, revealed significant differences between the “non-rescued” *pdrm/UAS-GFP* flies and the appropriate controls (*UAS-GFP/+* or *8.142/UAS-GFP, p <* 0.002); similarly, *pdrm/UAS-lacZ* flies are significantly different from their controls (*UAS-lacZ/+* and *8.142/UAS-lacZ, p <* 0.002). Pairwise comparisons revealed no significant differences between “rescued” *pdrm/UAS-Lmo^1^* flies and their “normal” controls (*8.142/UAS-Lmo^1^* and *UAS-Lmo^1^/+, p* = 0.99 and *p* = 0.14, respectively) or *pdrm/UAS-Lmo^2^* flies and their controls (*8.142/UAS-Lmo^2^/+* and *UAS-Lmo^2^/+, p* = 0.09 and *p* = 0.99, respectively), indicating full rescue of *pdrm* cocaine sensitivity. For all genotypes, *n* = 16–20 experiments.

The *Lmo* locus produces at least three transcripts by differential promoter use (Figure 3A). The RA and RC transcripts differ only in their 5′ UTRs and are predicted to encode identical proteins of 313 amino acids; the RB transcript is predicted to utilize an alternative translational start site in its first exon, which would result in an addition of 71 N-terminal amino acids. The insertions that produce increased cocaine sensitivity all lie within the putative promoter region for the RA transcript, 25–90 bp upstream of its transcriptional start site (Figure 3A). In order to identify molecular changes caused by these insertions, we assessed *Lmo* transcript levels by quantitative RT-PCR in wild-type flies and *Lmo* mutants. In wild-type flies the RA transcript was about 4-fold more abundant in heads as compared to bodies, suggesting that this transcript is enriched in the nervous system (Figure 3B; see also Figure 4). Importantly, the RA transcript was reduced by greater than 50% in the heads, but not the bodies, of *EP1306* flies (Figure 3B); a 40% increase in the RA transcript was observed in the heads of *Bx^J^* flies (data not shown). Thus, as predicted by the location of the P-element insertion, the *EP1306* mutation causes a reduction in *Lmo* transcript levels, a finding that is consistent with the observation that the cocaine sensitivity of *EP1306* flies is similar to that seen with known loss-of-function alleles of *Lmo* (the *hdp* alleles) and opposite to that seen with gain-of-function *Bx* alleles. In addition, the finding that transcript levels are specifically reduced in the heads of *EP1306* flies, and not their bodies, suggests that this mutation affects a nervous-system-enriched (or -specific) *Lmo* transcript (see below). Finally, none of the P-element insertions in the promoter of the RA transcript leads to a held-up wing phenotype, which is characteristic of loss-of-function *hdp* alleles (Shoresh et al. 1998; Milan and Cohen 1999). This suggests that the P-element insertions isolated in our genetic screen cause either less severe or more spatially restricted changes in *Lmo* gene expression.

### Restricted Expression of *Lmo* in the Nervous System Is Sufficient for Wild-Type Cocaine Sensitivity

The *pdrm* P[GAL4] insertion, which acts as a promoter/enhancer detector, is expected to reproduce, at least in part, the expression pattern of the *Lmo* gene. In adult flies, the *pdrm* line showed extensive GAL4 expression in the brain as visualized with the *UAS–green fluorescent protein (GFP)* transgene (Figure 4A). Prominent expression was observed in the antennal lobes (ALs) and the Kenyon cells of the mushroom bodies (MBs), which are major brain centers involved in olfaction and olfactory conditioning, respectively (Stocker 1994; Zars 2000). In addition, GAL4 was expressed in the LN_v_s, which are the major pacemaker cells regulating circadian locomotor rhythmicity in flies (Renn et al. 1999). Finally, GAL4 expression was seen in neurosecretory cells located in the pars intercerebralis, in glial cells surrounding the optic lobes, and in scattered cells throughout the ventral nerve cord (VNC) (Figure 4A; data not shown). In *pdrm* larvae and adult flies, GAL4 expression was restricted to the nervous system. GAL4 expression was not detected in larval imaginal discs, such as the wing disc (data not shown), where *Lmo* expression has been shown to play an important patterning role (Milan and Cohen 1999). Thus, *pdrm* traps a specific subset of *Lmo* regulatory elements, possibly those controlling nervous-system-restricted expression of the RA transcript.

We hypothesized that the expression pattern of the *pdrm* enhancer trap may identify the cells in which *Lmo* expression is disrupted in the P-element insertion lines, causing increased cocaine sensitivity. If this is the case, *pdrm*-driven expression of *UAS-Lmo* transgenes is expected to restore normal cocaine sensitivity to *pdrm* flies. Indeed, we found that two *UAS-Lmo* transgenes rescued the cocaine-sensitivity defect of *pdrm* mutant flies (Figure 4B). This effect was specific to *Lmo,* as *pdrm*-driven expression of *UAS-tauGFP* or *UAS-lacZ* failed to restore normal behavior. Finally, *Lmo* mutants that did not contain a GAL4 enhancer trap, such as *EP1306,* failed to be rescued by the presence of *UAS-Lmo* transgenes (data not shown). These data demonstrate that the cocaine-sensitivity defect of *pdrm* flies is due to the loss of *Lmo* function, and that nervous-system-specific expression of *Lmo* in cells dictated by the *pdrm* GAL4 line is sufficient to confer normal cocaine responses.

### *Lmo* Expression in PDF Neurons Is Sufficient to Confer Normal Cocaine Responses

In order to refine further the spatial requirements for *Lmo* function, we attempted to rescue the *EP1306* phenotype by expression of *Lmo* in specific brain regions. EP lines carry a P-element insertion containing multiple UAS sites to which GAL4 can bind to drive expression of adjacent genomic sequences (Rørth 1996). Therefore, mutagenic EP insertions, if oriented appropriately, can be used to drive expression of the disrupted gene. It was demonstrated previously that GAL4-driven *Lmo* expression can be mediated by the *EP1306* and *EP1383* insertions (Milan et al. 1998; Zeng et al. 1998). Consistent with this, we were able to rescue the *EP1306* phenotype in the presence of the heat-inducible *hs-GAL4* transgene, which drives low levels of ubiquitous GAL4 expression even in the absence of heat shock (data not shown).

**Figure 5:**
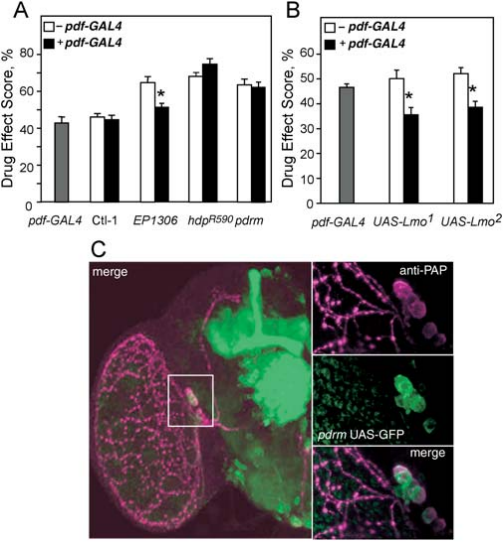
*Lmo* Expression in PDF Neurons Regulates Cocaine Responses (A) *pdf-GAL4*-driven expression of *Lmo* using the *EP1306* element rescues the *EP1306* insertional phenotype. *Lmo* mutants *EP1306, hdp,* and *pdrm,* as well as a control EP line (Ctl-1 *= EP1413*), were tested in the absence (−*pdf-GAL4;* white bars) and presence (***+*** *pdf-GAL4;* black bars) of *pdf-GAL4*. All male flies are hemizygous for the *Lmo* mutation (or Ctl-1) and heterozygous for *pdf-GAL4* (when carrying the transgene). One-way ANOVAs with post hoc planned comparisons (critical *p-*value adjusted to 0.0125) confirmed that *EP1306, pdrm,* and *hdp* flies (in the absence of *pdf-GAL4*) had significantly increased sensitivity to cocaine compared to control flies (Ctl-1 *= EP1413*) (*p* ≤ 0.003, *n* = 15–26 experiments). One-way ANOVA with post hoc planned comparisons (critical *p-*value adjusted to 0.01) revealed a significant difference between *pdrm/pdf-GAL4* and *hdp/pdf-GAL4* flies and their controls (*EP1413/pdf-GAL4, pdf-GAL4/+,* or *EP1413/+, p <* 0.003, *n* = 24–27 experiments), showing that the presence of *pdf-GAL4* does not rescue the cocaine sensitivity of *pdrm* or *hdp* flies. In contrast, similar comparisons for “rescued” *EP1306/pdf-GAL4* flies revealed no significant differences from their “normal” controls (*p* ≥ 0.026, *n* = 27–36). Furthermore, within-group comparisons (**+**/− *pdf-GAL4*) using *t*-tests indicate a significant difference only in the *EP1306* group (*p* = 0.002). Asterisk denotes significant difference between −*pdf*-GAL4 and **+** *pdf*-GAL4 phenotype. (B) Flies overexpressing *Lmo* in PDF cells show decreased sensitivity to cocaine. Flies heterozygous for both *pdf-GAL4* and either one of two *UAS-Lmo* transgenes (black bars) were compared to flies carrying *UAS-Lmo* (white bars) or *pdf-GAL4* (gray bar) alone. One-way ANOVA revealed a significant effect of genotype for both *UAS-Lmo* transgene groups. Post-test planned comparisons, with the critical *p*-value adjusted to 0.025, showed significant differences between the *pdf-GAL4/UAS-Lmo* flies and either *pdf-GAL4/+* (*p <* 0.02) or *UAS-Lmo/+* controls (*p <* 0.005). Asterisks denote significant differences*, n* = 16 experiments. (C) Confocal images demonstrate overlap between *pdrm* and PDF expression in the LN_v_s. In the left panel, *UAS-mCD8GFP* reveals the *pdrm*-driven GAL4 expression pattern (green) in the adult brain, and α-PAP staining (magenta) reveals PDF-expressing LN_v_s. Right panels are close-ups of the cell bodies of the LN_v_s; white areas correspond to regions of overlap between GFP (green) and PAP (magenta) expression.

We then used GAL4 enhancer-trap lines with expression in specific brain regions to drive *Lmo* expression from the *EP1306* insertion. We focused specifically on GAL4 lines that drive expression in the MBs, ALs, pars intercerebralis neurons, LN_v_s, and glia, sites of expression revealed by the *pdrm* line (Figure 4A). GAL4 lines that drive expression specifically in the MBs and the ALs failed to rescue the cocaine-sensitivity phenotype of *EP1306* (data not shown). Similar negative results were obtained with the glial-specific *repo-GAL4* driver (Xiong et al. 1994). However, expression of *Lmo* in the LN_v_s using the *pdf-GAL4* driver, which drives expression in cells that contain the neuropeptide PDF (Renn et al. 1999), restored nearly wild-type cocaine responsiveness to *EP1306* flies (Figure 5A and 5C). *pdf-GAL4* did not rescue the cocaine phenotype of *hdp* and *pdrm* flies, demonstrating dependence on induced *Lmo* expression provided by the *EP1306* insertion. These results show that *Lmo* expression in the PDF-expressing neurons alone is sufficient to rescue wild-type cocaine responses, implicating these cells in the increased cocaine responses observed in *Lmo* loss-of-function mutants.

In order to test whether LN_v_s are also the locus for the resistance to cocaine observed in *Bx* mutants, we overexpressed *Lmo* in wild-type flies using the *pdf-GAL4* driver and *UAS-Lmo* transgenes. We found that these flies showed a significant decrease in cocaine sensitivity (Figure 5B). The magnitude of the induced resistance was lower than that observed in the *Bx^J^* strain, but similar to that of weaker *Bx* alleles. It is possible that overexpression of *Lmo* with the *pdf-GAL4* driver may not be as high as that caused by the *Bx^J^* mutation, or, alternatively, that overexpression of *Lmo* in cells other than the LN_v_s mediates the remaining resistance to cocaine observed in *Bx^J^*. Nonetheless, this experiment supports a role for LN_v_s as a site where *Lmo* regulates sensitivity to cocaine.

In the adult fly, *pdf*-*GAL4* drives expression in small and large LN_v_s (s-LN_v_s and l-LN_v_s) and in a group of neuroendocrine cells located at the very tip of the VNC (J. H. Park et al. 2000; Figure 5C). The LN_v_s express many central clock genes and have been demonstrated, through genetic ablations and electrical silencing studies, to play a central role in maintaining circadian locomotor rhythmicity (Renn et al. 1999; Nitabach et al. 2002; Peng et al. 2003); the function of the *pdf-GAL4-*expressing cells in the VNC is unknown. The *pdrm* line drives expression in both the s-LN_v_s and l-LN_v_s, as demonstrated by immunohistochemical analysis of *pdrm/UAS-GFP* flies with antibodies directed against the PDF precursor PAP (Figure 5C). *pdrm* does not, however, drive expression in the PDF-expressing cells located in the VNC (data not shown). This overlap in expression of *pdrm* and *pdf-GAL4* in the LN_v_s implicates these few neurons as mediators of the altered cocaine sensitivity observed in *Lmo* mutants.

Because of the known role of LMOs as regulators of developmentally important transcription factors (Hobert and Westphal 2000), we asked whether the development of PDF-expressing LN_v_s is disrupted in *Lmo* mutants. Projections of the s-LN_v_s and l-LN_v_s can be visualized with an antibody that recognizes the PDF precursor PAP (Renn et al. 1999). L-LN_v_s make an elaborate network of varicosities on the surface of the optic medulla, and project across the midline to the contralateral LN_v_s, while s-LN_v_s make a very specific projection to the dorsal central brain (Figure 5C). Both sets of LN_v_ neurons also make extensive arborizations in the accessory medulla (reviewed in Hellfrich-Förster 2003). In the *EP1306* and *pdrm* mutants, anti-PAP staining revealed that the number and detailed morphology of LN_v_ neurons were completely normal (data not shown). Thus, *Lmo* does not appear to play a role in the development of the LN_v_s, although subtle developmental defects could have been missed.

### *Lmo* Is Required for Robust Circadian Locomotor Rhythms

**Figure 6:**
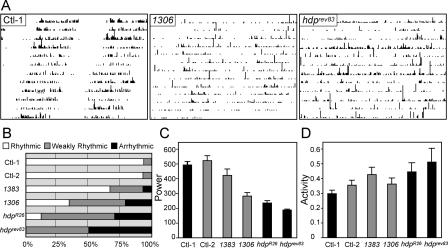
Wild-Type *Lmo* Is Required for Robust Circadian Rhythms of Locomotor Activity Locomotor activity of control (Ctl-1 and Ctl-2) and *Lmo* mutant (*EP1383, EP1306, hdp^R26^,* and *hdp^rev83^*) flies was recorded in constant darkness as previously described (Nitabach et al. 2002). (A) Representative actograms of control and *Lmo* mutants. Control flies show robust circadian rhythms with clear distinctions between activity during the subjective day and inactivity during the subjective night. The pattern of the *EP1306* and *hdp^rev83^* mutants was more stochastic. (B) Graph showing the proportion of strongly rhythmic (white), weakly rhythmic (gray), and arrhythmic (black) flies for each genotype. Most control flies had strong rhythms (28/30 for Ctl-1 and 27/29 for Ctl-2) while *Lmo* mutants formed a series with an increasing fraction of the flies arrhythmic. For example, 13/26 *hdp^rev83^* flies were arrhythmic, with the other 13 all having weak rhythms. The power of the rhythm was used to estimate the strength of the activity rhythm, with a power of 300 or more classed as a strong rhythm and a power between 300 and 170 classed as a weak rhythm; arrhythmics were given a power of 170 (for analysis below). Between 24 and 30 flies were assayed for each genotype. There were no major differences in the period length of the rhythmic flies in each genotype (Ctl-1, 23.6 ± 0.3; Ctl-2, 23.5 ± 0.5; *EP1383,* 23.2 ± 0.4; *EP1306,* 23.4 ± 0.3; *hdp^R26^,* 24.2 ± 0.5; and *hdp^rev83^,* 23.4 ± 0.3). (C) Quantitation of the average power of the rhythm with error bars showing standard error of the mean. One-way ANOVA revealed significant differences between genotypes (*p <* 0.0001). Post-hoc *t*-tests using a Bonferroni correction revealed that the power of the rhythm was significantly different between control flies and the *Lmo* mutants *hdp^R26^, hdp^rev83^,* and *EP1306* (*p <* 0.01). *EP1383* flies had a significantly weaker rhythm than Ctl-2 flies (*p <* 0.05). Ctl-1, *w^1118^,* is the appropriate genetic control for the *hdp* alleles (black columns); Ctl-2, *EP1631,* is the appropriate control for *EP1306* and *EP1383* (gray columns). All flies tested are in the same genetic background, that of the *w^1118^* flies. (D) Quantitation of average activity (beam crossings per minute) with error bars showing standard error of the mean. ANOVA did not reveal significant differences between genotypes at the 0.01 level. Ctl-1, *w^1118^,* is the appropriate genetic control for the *hdp* alleles (black columns); Ctl-2, *EP1631,* is the appropriate control for *EP1306* and *EP1383* (gray columns). All flies tested are in the same genetic background, that of the *w^1118^* flies.

The increased sensitivity to cocaine of *Lmo* mutants suggested that these mutations alter LN_v_ function without grossly affecting LN_v_ development. In order to determine whether *Lmo* mutants have a more general dysfunction in the LN_v_s, we tested *Lmo* mutants in locomotor rhythm assays. The LN_v_s are required for the maintenance of circadian locomotor rhythms in constant darkness, and are thus the pacemaker neurons of the fly (Stanewsky 2003). *Lmo* mutants *EP1306, EP1383, hdp^R26^,* and *hdp^rev83^* had less robust locomotor rhythms in constant darkness than their control lines (Figure 6A; data not shown), and all alleles had a higher tendency for arrhythmicity (Figure 6B). We used the power of the rhythm as an estimate of the degree of rhythmicity and found that *EP1306* flies had weaker behavioral rhythms than *EP1383* flies, which, in turn, were less robustly rhythmic than their control line (Ctl-2; Figure 6C); similarly, *hdp^R26^* and *hdp^rev83^* flies had significantly weaker rhythms than their control line (Ctl-1; Figure 6C). However, there were rhythmic flies in almost all genotypes assayed (Figure 6B), and the period lengths of the rhythms were very similar across the genotypes (see legend to Figure 6 for details). Together, these data demonstrate that disruption of *Lmo* results in weak circadian rhythms of behavior and suggest a generalized dysfunction of the LN_v_s in *Lmo* mutants.

### LN_v_s Regulate Circadian Rhythmicity and Cocaine Sensitivity Independently

**Figure 7:**
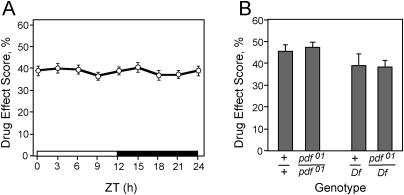
Cocaine Responses Are Not a Circadian Output and *pdf* Mutants Show Wild-Type Cocaine Sensitivity (A) Cocaine responses do not vary with the circadian clock. Control *(EP1631)* flies were raised under LD conditions and assayed for cocaine phenotypes in the crackometer at the indicated Zeitgeber (ZT) times. One-way ANOVA revealed no significant effect of time of day (*F* = 0.53, *p* = 0.82, *n* = 32). (B) Flies lacking the neuropeptide PDF *(pdf^01^)* display normal cocaine sensitivity. *pdf^01^* homozygotes *(pdf^01^/pdf^01^)* and *pdf^01^* hemizygotes *(pdf^01^/Df)* showed wild-type responses to cocaine in the crackometer. Individual pairwise comparisons using Student's *t*-tests revealed no significant differences between control (+/+ and +/*Df*) and *pdf* mutant genotypes (*p* = 0.69, *p* = 0.97, *n* = 6–8 experiments)

The observation that *Lmo* functions in the PDF neurons to regulate cocaine sensitivity, together with the finding that *Lmo* mutants show weak circadian locomotor rhythms, suggested that the pathways regulating cocaine sensitivity interact with the circadian clock. Alternatively, *Lmo* could regulate these two behaviors independently. We addressed these possibilities in two sets of experiments. First, we determined whether cocaine sensitivity was regulated by the circadian clock. For this purpose we entrained flies to light–dark (LD) cycles and then tested them for cocaine sensitivity during the light and dark phases at 3-h intervals over 24 h (see Materials and Methods). We found that cocaine sensitivity was essentially the same at all times tested (Figure 7A), demonstrating that cocaine responsiveness is not a behavioral output of the circadian clock.

Second, we asked whether PDF, the only known functional output of the LN_v_s in the context of circadian rhythms, is involved in regulating cocaine sensitivity. *pdf* mutant flies show a circadian phenotype that has been localized to the LN_v_s. We found that the *pdf^01^* mutant flies, which completely lack PDF (Renn et al. 1999), showed normal cocaine responses compared to the control strain (Figure 7B). To eliminate the possibility that the absence of a phenotype was caused by genetic modifiers in the background of the *pdf^01^* strain, we also tested flies carrying the *pdf^01^* chromosome over a deficiency for the locus (see Materials and Methods). These *pdf^01^* hemizygous flies also displayed normal cocaine sensitivity (Figure 7B). Taken together, these results show that the altered cocaine sensitivity of *Lmo* flies is not secondary to their abnormal circadian rhythms. Moreover, our data show that cocaine sensitivity and circadian behaviors, although both localized to the LN_v_s, are genetically separable.

### LN_v_s and Their Synaptic Activity Regulate Acute Cocaine Responsivity

**Figure 8:**
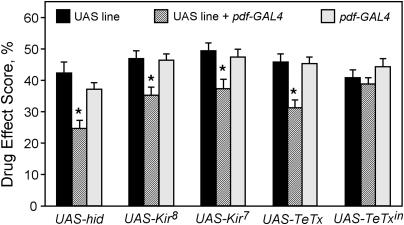
Silencing or Ablating PDF Cells Induces Resistance to Cocaine Ablation of PDF cells with *pdf-GAL4* and *UAS-hid* reduced sensitivity to cocaine compared to parental lines (*pdf-GAL4/+* and *UAS-hid/+*). Electrical *(UAS-Kir2.1^8^* or *UAS-Kir2.1^7^)* or synaptic *(UAS-TeTx)* silencing of PDF cells with *pdf*-*GAL4* phenocopied PDF cell ablations. One-way ANOVAs with post hoc planned comparisons revealed a significant effect of genotype for the *UAS-hid* (*p <* 0.003, *n* = 20), *UAS-Kir2.1^7^* (*p <* 0.008, *n* = 32), *UAS-Kir2.1^8^* (*p <* 0.002, *n* = 28), and *UAS-TeTx* (*p <* 0.001, *n* = 28) groups, but not for *UAS-TeTx^in^* (*p* > 0.045, *n* = 28) (*n* corresponds to the number of experiments). Critical *p*-value was adjusted to *p* = 0.025. Asterisks denote significant differences in both planned comparisons (*pdf-GAL4/+* and *UAS-*transgene/+ versus *pdf-GAL4/UAS*-transgene). Variations in phenotype of *pdf-GAL4* flies for each set of experiments is caused by day-to-day variability.

The hypersensitivity of *Lmo* mutants to cocaine could result from either the disruption of an LN_v_ output that acts normally to dampen cocaine sensitivity or from an increase in an output of LN_v_s that normally enhances cocaine sensitivity. In order to differentiate between these two possibilities, we tested flies that lacked LN_v_s, generated by targeted expression of the cell death gene *head involution defective (hid)* using *pdf-GAL4*. This approach was previously used to demonstrate that LN_v_s are the *Drosophila* pacemaker neurons responsible for rhythmic locomotor activity (Renn et al. 1999). When tested in the crackometer, flies with LN_v_ ablations showed reduced sensitivity to cocaine when compared to controls (Figure 8). These results show that LN_v_s normally act to increase cocaine responsiveness and that this effect is antagonized by *Lmo* function in these cells (see Discussion).

To confirm that the behavioral resistance observed in LN_v_-ablated animals results from loss of neuronal signaling from these cells, rather than from developmental compensations induced by their ablation, we functionally silenced the LN_v_s either electrically, by targeted expression of the mammalian inward rectifying K^+^ channel *(Kir2.1),* or synaptically, by expression of tetanus toxin light chain *(TeTx)* (Sweeney et al. 1995; Baines et al. 2001). These manipulations do not affect LN_v_ survival or the normal projection patterns of LN_v_s (Kaneko et al. 2000; Nitabach et al. 2002). Expression of either *Kir2.1* or *TeTx* resulted in reduced cocaine sensitivity similar to that seen with LN_v_ ablations (Figure 8). Importantly, expression of an inactive *TeTx (TeTx^in^)* did not significantly alter cocaine responses, confirming that the actions of these transgenes were specific to their ability to silence or ablate the LN_v_s (Figure 8). These data further demonstrate that circadian phenotype does not predict cocaine phenotype, as targeted expression of either *hid* or *Kir2.1* in LN_v_s causes arrhythmia, while expression of *TetTx* does not (Renn et al. 1999; Kaneko et al. 2000; Nitabach et al. 2002). In summary, these results confirm a novel role for LN_v_ activity and synaptic output in increasing behavioral responses to cocaine in a manner independent from its role in regulating circadian locomotor rhythmicity.

## Discussion

In a genetic screen for *Drosophila* mutants with altered acute responses to cocaine, we isolated multiple mutations in the *Lmo* locus. Behavioral characterization of gain- and loss-of-function alleles of *Lmo* demonstrates an inverse correlation between *Lmo* expression levels and cocaine sensitivity. Through targeted expression of *Lmo,* we show that these altered cocaine responses are caused by differential *Lmo* expression in the circadian pacemaker neurons, the LN_v_s. Consistent with a dysfunction of these pacemaker neurons in *Lmo* mutants is our finding that the mutant flies also show altered circadian locomotor rhythms. However, using a variety of genetic methods to ablate or functionally silence these neurons, we reveal a novel role for the LN_v_s in modulating cocaine's acute locomotor responses that is independent of their pacemaker function. These findings add to mounting data in *Drosophila* and mice supporting a role for circadian genes in cocaine-related behaviors (Andretic et al. 1999; S. K. Park et al. 2000; Abarca et al. 2002; Rothenfluh and Heberlein 2002). Our discovery that cocaine actions are modulated by neurons critical for normal circadian locomotor rhythmicity suggests a basis for this overlap.

### *Lmo* Functions in PDF Neurons to Regulate Cocaine Sensitivity

We provide several lines of evidence that levels of *Lmo* expression in PDF-expressing LN_v_s regulate acute sensitivity to volatilized cocaine in *Drosophila*. First, loss-of-function mutations in *Lmo* show increased cocaine sensitivity, a defect that can be reversed by induced expression of *Lmo* in the LN_v_s. Second, *Bx* mutants, in which *Lmo* is overexpressed, show reduced cocaine sensitivity; this resistance can be mimicked by overexpression of *Lmo* in the LN_v_s. The LN_v_s are a group of 8–10 neurons in each brain hemisphere that express the neuropeptide PDF. These neurons have previously been identified as the circadian pacemaker neurons of adult *Drosophila* and are involved in modulating rhythmic locomotor behavior (Renn et al. 1999; Blanchardon et al. 2001). Interestingly, expression of a mouse homolog of *Lmo, Lmo4,* is highly enriched in the suprachiasmatic nucleus (SCN) (A. Lasek, D. Kapfhamer, and U. H., unpublished data), the mammalian central pacemaker. Furthermore, microarray analysis revealed that in many tissues, *Lmo4* expression varies with circadian time (Panda et al. 2002). These data suggest an evolutionarily conserved role for *Lmo* in clock neuron function.

LMOs are known to act as regulators of LIM-HD protein activity and stability (Retaux and Bachy 2002). LIM-HD proteins are transcription factors involved in many stages of nervous system development, from neuronal generation and axon guidance to determination of neuronal subtype identity (Hobert and Westphal 2000). In addition, expression of LIM-HD proteins in postmitotic neurons suggests a role in maintaining the differentiated state of these neurons (Hobert and Westphal 2000). The development and structure of the PDF-expressing LN_v_s, the neurons to which we localize *Lmo* action, have been studied in detail (Helfrich-Förster 1997). The LN_v_s can be divided into two groups of 4–5 neurons based on cell body size, projection pattern, and time of development. The s-LN_v_s, which arise in early larval development, project to the dorsal central brain, terminating near two sets of dorsal neurons that express clock genes at high levels. The l-LN_v_s arise during pupal stages and project onto the surface of the optic medulla, as well as to the contralateral LN_v_s through fibers running in the posterior optic tract. Both s- and l-LN_v_s have dense arborizations in the accessory medulla, a neuropil proposed to be a circadian pacemaker center in cockroaches and crickets (Helfrich-Förster 1998). An assessment of s-LN_v_ and l-LN_v_ numbers and detailed projection patterns revealed no differences between wild-type flies and *Lmo* mutants. Furthermore, by both quantitative RT-PCR and immunohistochemistry, we determined that PDF levels are normal in *Lmo* mutants (data not shown). Expression of PDF, the only known LN_v_ output, is restricted to the LN_v_s and a few tritocerebral neurons. As PDF expression is a specific marker for the differentiated state of the LN_v_s, it is unlikely that in *Lmo* mutants these neurons are grossly abnormal in their terminal differentiation.

The absence of obvious structural abnormalities of LN_v_s suggests that *Lmo* may play an active role in regulating cocaine responses. In fact, evidence is mounting that the expression and activity of LMOs are dynamically regulated within the nervous system. For instance, expression of the murine *Lmo* homologs *Lmo1, Lmo2,* and *Lmo3* is differentially regulated by seizure activity in specific regions of the hippocampus and forebrain of adult mice (Hinks et al. 1997). In addition, gene array experiments have revealed that expression of mammalian *Lmo* homologs is under circadian regulation in the SCN and is increased in the cerebral cortex during sleep deprivation (Cirelli and Tononi 2000; Panda et al. 2002). Furthermore, *Lmo3* was isolated as a transcript upregulated by DA administration in cultured astrocytes (Shi et al. 2001). Lastly, *Lmo2* and *Lmo4* were recently isolated in a screen for calcium-regulated activators of transcription (Aizawa et al. 2004), suggesting a role for LMOs in regulating gene expression changes induced by neural activity. These data are intriguing in light of the many functional changes that occur in reward pathways in the addicted state. Whether LMO activity and/or expression are regulated by acute cocaine exposure remains to be studied.

### Separable Roles of LN_v_s in Regulating Cocaine and Circadian Behaviors

We provide evidence that the LN_v_s regulate cocaine-induced behaviors, in addition to their well-known role in controlling circadian locomotor behaviors. Flies with LN_v_ ablations show reduced sensitivity to cocaine, establishing that these cells normally increase behavioral responses to cocaine. Our experiments also indicate that LN_v_s drive circadian locomotor and cocaine behaviors in distinct ways. First, we found that sensitivity to cocaine is not modulated in a circadian manner, showing that the cocaine response is not simply an output of the central clock. Second, we showed that *Lmo* mutants, *pdf* mutants, and flies in which LN_v_s have been electrically silenced or ablated—all known to display similar locomotor rhythm deficits—show completely uncorrelated cocaine sensitivities: increased, unchanged, and reduced sensitivity, respectively. Lastly, we provide evidence that the LN_v_ outputs that mediate cocaine and circadian behaviors are divergent. PDF, the only known LN_v_ neurotransmitter, is required for normal locomotor rhythms (Renn et al. 1999). *pdf* null mutants, however, show normal cocaine responses. Furthermore, while synaptic silencing of PDF neurons does not disrupt circadian locomotor activity (Kaneko et al. 2000), we show here that the same manipulation reduces cocaine responses to the same extent as neuronal ablation. Together, these results imply the existence of an alternate, TeTx-sensitive functional output that mediates LN_v_ modulation of cocaine responses. Interestingly, Blau and colleagues have also hypothesized a PDF-independent LN_v_ output that regulates another rapid behavioral response, larval photophobicity (E. Mazzoni, C. Desplan, and J. Blau, unpublished data).

How might LN_v_s modulate circadian rhythmicity and cocaine sensitivity independently? It is possible that these cells use distinct output mechanisms, PDF to regulate circadian locomotor rhythms and another unknown signal to regulate cocaine sensitivity. Alternatively, these behaviors could be regulated by distinct subsets of LN_v_s. For example, several recent findings suggest that l-LN_v_s may play a lesser role in regulating circadian rhythmicity (Helfrich-Förster 2003). First, l-LN_v_s do not project to the dorsal brain, an area implicated in locomotor rhythmicity (Helfrich-Förster 1997). Second, unlike in s-LN_v_s, molecular clock cycling is not sustained for long in these cells during free-running conditions (Kaneko et al. 2000; Yang and Sehgal 2001; Shafer et al. 2002). Lastly, PDF expression and release is modulated by the molecular clock in s-LN_v_s, but not in l-LN_v_s (Blau and Young 1999; J. H. Park et al. 2000). However, another study found that normal rhythmicity can be obtained in flies lacking s-LN_v_s (Helfrich-Förster 1998). Moreover, the projections of l-LN_v_s connect the l- and s-LN_v_s from both brain hemispheres through projections in the posterior optic tract, suggesting a functional link between the two groups of cells. Whether *Lmo* functions in the s- and/or l-LN_v_s to regulate cocaine sensitivity and circadian rhythmicity cannot be established with currently available tools.

There is growing evidence for a functional link between circadian neurons and the modulation of cocaine-related behaviors. In mammals, the peptidergic neurons of the SCN have been shown by a variety of studies to be the central pacemakers controlling circadian rhythms (van Esseveldt et al. 2000). Interestingly, the fetal mammalian SCN contains DA D1 receptors through which cocaine can influence entrainment of fetal biological rhythms (Simonik et al. 1994; Viswanathan et al. 1994; Bender et al. 1997). In addition, disruption of another major component of the mammalian circadian system, the pineal gland, or its secretory product melatonin, results in altered cocaine responses (Uz et al. 2002, 2003; Zhdanova and Giorgetti 2002). Hirsh and colleagues showed that mutants in the *Drosophila* clock genes *period, clock,* and *cycle,* but not *timeless* fail to sensitize to repeated cocaine exposures (Andretic et al. 1999). These genes, which are expressed at high levels in LN_v_s, have been to shown to act in these cells to modulate circadian behavior (Kaneko et al. 1997). It is not known whether circadian gene function in LN_v_s also regulates behavioral sensitization to cocaine.

### How Might LN_v_s Modulate Cocaine Responses

**Figure 9:**
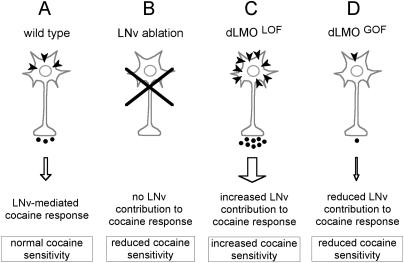
A Model for LN_v_ and LMO Regulation of Cocaine Sensitivity (A) In wild type, LN_v_s modulate locomotor responses via electrical activity and synaptic transmission. We propose a model in which cocaine acts to directly increase LN_v_ activity. Upon cocaine administration, synaptic DA concentrations are increased (via cocaine's inhibition of the plasma membrane DA transporter). Activation of presumed DA receptors on the LN_v_ (dark arrowheads) stimulates electrical activity and subsequent synaptic output. This activity contributes to the behavioral response of the fly to cocaine. (B) LN_v_ ablations eliminate LN_v_ contribution to the cocaine response, reducing cocaine sensitivity. (C) In our model, *Lmo* loss-of-function mutants *(Lmo^LOF^),* which have increased cocaine sensitivity, have increased activity/output during the cocaine response. This increased activity may be mediated by increases in receptor content on the LN_v_ or by recruitment of other LN_v_s that normally do not participate in the cocaine response. (D) *Lmo* gain-of-function mutants *(Lmo^GOF^)* mutants have reduced LN_v_ output and reduced cocaine sensitivity. This could also result from a reduction in receptor density.

We have demonstrated that LN_v_ electrical activity and synaptic output contribute to cocaine-induced behavioral responses, raising a number of questions regarding the interaction of cocaine with these neurons and their output. We propose a simple model whereby the activity of some or all LN_v_s directly increases upon cocaine administration, which in turn results in cocaine-induced changes in locomotion (Figure 9). Interestingly, a recent report showed that cultured LN_v_ neurons can respond to either DA or acetylcholine, but not to glutamate, serotonin, octopamine, or histamine (Wegener et al. 2004). The inferred presence of DA receptors on a subset of LN_v_s provides a potential mechanism by which LN_v_ activity could be directly increased by cocaine administration, as cocaine's primary mechanism of action is to inhibit the reuptake of DA by DAT. In this model, when the LN_v_s are ablated or silenced, one site of cocaine action would be eliminated, thus reducing cocaine's effect (Figure 9B).

How would *Lmo* fit into this model? We propose that in *Lmo* loss-of-function mutants, LN_v_ output is boosted, possibly because of increased expression of DA receptors (Figure 9C), leading to enhanced cocaine sensitivity. Conversely, increased *Lmo* expression (in *Bx* mutants or in flies specifically overexpressing *Lmo* in PDF cells) would result in reduced receptor content and, consequently, in dampened cocaine sensitivity (Figure 9D). Further studies are needed to identify the putative DA receptor (or other molecules) that functions in LN_v_s to regulate cocaine-induced behaviors.

LMO-induced changes in receptor expression are not inconceivable given LMO's interaction with LIM-HD proteins. Changes in LIM-HD protein function have been shown to affect aspects of neuronal subtype identity, including neurotransmitter and receptor expression profiles. For instance, mutations in the *Drosophila* LIM-HD gene *islet* cause a loss of DA and serotonin synthesis, while ectopic expression leads to ectopic expression of tyrosine hydroxylase, an enzyme required for DA biosynthesis (Thor and Thomas 1997). In addition, expression of a *Drosophila* DA receptor in larval neurons requires the function of the LIM-HD gene *apterous* (Park et al. 2004). The observation that DA receptor expression is also altered in various Caenorhabditis elegans LIM-HD mutants (Tsalik et al. 2003) suggests an evolutionarily conserved role of LIM-HD proteins and possibly LMOs in the regulation of neurotransmitter identity and responsiveness. Consistent with this idea is the finding that *Lmo* homologs are highly expressed in the mesolimbic DA system of mice (A. Lasek, D. Kapfhamer, and U. H., unpublished observations)—a neural pathway involved in the acute stimulant and rewarding properties of abused drugs. We posit that LMOs are ideally suited to modulate in a subtle manner the neurochemical identity and sensitivity of the nervous system to various stimuli, including drugs of abuse.

## Materials and Methods

### 

#### *Drosophila* culture and strains.

All flies were maintained on standard cornmeal molasses agar at 25 °C and 70% humidity under constant weak light. The Rørth EP collection was obtained from G. M. Rubin (University of California, Berkeley, California, United States) (Rørth 1996). The *pdrm* allele was originally isolated in a genetic screen of P[GAL4] insertions (carrying the GawB element) for altered response to the locomotor-activating effects of ethanol (F. Wolf, unpublished data) and consequently tested for cocaine sensitivity. The location of the insertion was determined by inverse PCR (http://www.fruitfly.org/about/methods/inverse.pcr.html). The *hdp^R590^* and *hdp^R26^* loss-of-function alleles were provided by S. Cohen (European Molecular Biology Laboratory, Heidelberg, Germany) (Milan et al. 1998). *UAS-Lmo* lines were provided by C. Zeng (University of Wisconsin, Milwaukee, Wisconsin, United States) (Zeng et al. 1998). The *hdp^rev83^* strain was generated by N. Justice by imprecise excision of the *EP1306* line, screening for the *hdp* phenotype (unpublished data). *Bx* alleles and *pdf* deficiencies *(Df(3R)T1-X* and *Df(3R)T1-P)* were obtained from the *Drosophila* Stock Center (Bloomington, Indiana, United States). *w33, pdf^01^* and *pdf-GAL4* flies were obtained from P. Taghert (Washington University, St. Louis, Missouri, United States) (Renn et al. 1999). *UAS-Kir2.1* lines were obtained from S. Sweeney (University of California, San Francisco, California, United States) (Baines et al. 2001). *UAS-TeTx* lines were generated as previously described (Sweeney et al. 1995; Scholz et al. 2000). *UAS-hid* flies were provided by R. S. Stowers (NASA Ames Research Center, Moffett Field, California, United States) (Zhou et al. 1997). *UAS-tauGFP* and *UAS-lacZ* lines were obtained from Y.-N. Jan (University of California, San Francisco). All lines used for behavioral experiments, unless noted below**,** were out-crossed for five generations to a *w^1118^* stock isogenic for Chromosomes II and III. Because *pdf^01^* and its background control strain *(w33)* are unmarked, only Chromosomes I and II were replaced via crosses to balancer stocks in the *w^1118^* genetic background.

#### Genetic screen and selection of controls

The approximately 400 X-linked EP lines were initially screened as hemizygotes crossed to a *w, X^X/Y* tester strain in the crackometer as described below (*n* = 6–9). These lines distributed normally, and 30 lines with phenotypes greater than 1.5 standard deviation from the mean were out-crossed into our *w^1118^* background and retested. An additional 70 lines were selected to be out-crossed in order to provide a population distribution from which to select control lines. These 100 out-crossed lines were rescreened for acute responses to cocaine. A group of five control EP lines were chosen based on having a normal acute response (near the mode of the distribution). In all behavioral experiments involving EP lines, at least three of these control lines were tested to ensure that the control shown was representative of the EP population. The specific control EP line shown for each experiment is noted in the figure legends. As a control for the *pdrm* line we used a P[GAL4] insertion (line *8.142*) that shows a normal response to multiple drugs, including cocaine (A. Rothenfluh, D. Guarnieri, A. Rodan, and F. Wolf, unpublished data). For data shown in Figures 1 and 3, all lines were crossed to a *w, X^X/Y* tester strain to reduce possible effects of recessive autosomal modifiers. In addition to the genotypes shown, we tested three P-element lines in the *Lmo* locus that did not produce cocaine phenotypes: *MS1096,* which lies 20 bp downstream of the first RA transcript exon, and two additional EP lines, *EP0443* and *EP1394,* inserted 5′ to *EP1383;* the EP lines have normal wings. *MS1096* has been shown to have a very weak wing venation phenotype, but has otherwise not been phenotypically or molecularly characterized (Milan et al. 1998).

#### Behavioral assays and statistics

For cocaine-sensitivity assays, for all behavioral experiments, 15 male flies were collected under CO_2_ anesthesia 0–2 d post eclosion (day 12), and tested 2–3 d later. Flies were equilibrated to room temperature for at least 1 h before being exposed to cocaine. Cocaine exposures were performed as described previously (McClung and Hirsh 1998; Bainton et al. 2000), and startle-induced negative geotaxis was assayed in a glass cylinder as described previously (Bainton et al. 2000). A drug effect score was determined as the average (measured every minute over 5 min) number of flies that remained on the bottom of the cylinder, expressed as percent of the total number of flies. Significance was established for each experiment as described in figure legends. Generally, either Student's paired *t*-tests assuming equal variance or one-way ANOVAs with post hoc Bonferroni planned comparisons or Tukey-Kramer multiple comparisons were performed in GraphPad Prism 4 (GraphPad, San Diego, California, United States). Error bars in all experiments represent the standard error of the mean. To maintain an experiment-wide error rate of α = 0.05, the adjusted error rates were *p* = 0.05/*n* for the *n* subsequent planned pairwise comparisons in each experiment. To observe cocaine locomotor activity patterns, ten flies were exposed to volatilized cocaine for 1 min, transferred to a 7.5 cm × 10 cm × 0.5 cm acrylic box, and images were captured on an Apple G4 computer (Apple, Cupertino, California, United States) using Adobe Premiere (Adobe Systems, San Jose, California, United States) at 10 frames/s for 5 min. Fly locomotion was tracked using the dynamic image analysis system software (Solltech, Oakdale, Iowa, United States). All genotypes were tested at each dose multiple times (*n* ≥ 3), and representative data were selected for the figures. Baseline negative geotaxis was measured by mock exposures of flies and subsequent assay in the crackometer as above; all genotypes displayed behavioral scores of less than 10%, with no significant differences between genotypes (*n* > 8).

For circadian experiments, locomotor activity of individual flies was measured using the TriKinetics (Waltham, Massachusetts, United States) infrared beam-crossing system recording total crosses in 10-min bins. Raw activity histograms were analyzed for circadian rhythms using Actimetrics (Wilmette, Illinois, United States) Clocklab software. Chi-square periodograms were constructed according to Sokolove and Bushell (1978), and significant circadian rhythmicity was defined as presence of a peak in periodogram power that extends above the 0.01 Chi-prosquare significance line. Since this line is equal to a power of approximately 175 at a period of 24 h, flies with no periodogram peak crossing the significance line were assigned a circadian power of 170. This would tend to overestimate the circadian power of these flies, and thus is conservative with regard to assessing statistical differences in power between genotypes exhibiting frequent arrhythmicity and those that are predominately rhythmic. For the circadian cocaine-sensitivity experiment, flies were set up and raised in LD, collected under CO_2_ anesthesia during the light phase 1–2 d after eclosion, and placed back in LD for 2–3 d. At the indicated Zeitgeber times, flies were tested in the crackometer, under lights, within 5 min of being removed from LD conditions.

#### Histology

Expression pattern of the *pdrm* P[GAL4] insertion was examined by crossing to *UAS-GFPT2* and *UAS-mCD8GFP*. All adult and larval preparations were dissected in PBS, fixed in 4% formaldehyde/PEM for 40 min, washed in PBS, and dehydrated in 50% glycerol/PBS for 1 h. Tissue was mounted in Vectashield mounting medium (Vector Laboratories, Burlingame, California, United States) and analyzed with a Bio-Rad confocal microscope with Bio-Rad Lasersharp 2000 software (Bio-Rad, Hercules, California, United States). PAP antibody staining (provided by P. Taghert) was performed on CNS preparations that were dissected, fixed, and washed as above. Specimens were incubated in 1:2,000 dilution of anti-PAP in PBT, and with a Texas Red–coupled goat anti–guinea pig secondary antibody, diluted 1:200 (Jackson Laboratory, Bar Harbor, Maine, United States).

#### Real-time quantitative RT-PCR

Flies 2- to 4-d-old were collected and frozen immediately at −80 °C. Heads were removed from bodies by vortexing, and separated in a sieve. RNA was extracted from heads and bodies by homogenizing the flies in hot phenol and NTES. Complementary DNA (cDNA) was prepared using TaqMan Reverse Transcription Reagents (Applied Biosystems, Foster City, California, United States) according to the manufacturer's specifications, with the addition of random sequence hexamers to 2.5 μM. cDNA was analyzed by quantitative, real-time PCR using the ABI PRISM 7700 Sequence Detection System (Applied Biosystems). The following probes and primers were designed using ABI PrimerExpress software: *Lmo*RA-For, GAAGAGAAACAACAGCAGCAACA; *Lmo*RA-Rev, ATTTGCATATTTCGCACTTGTTTAGCT; and *Lmo*RA-Probe, CTGCTGCCGTTGCTG. *rp49* probe and primers (CT 6405) were obtained from Applied Biosystems. TaqMan PCR reactions consisted of 50 ng of cDNA, 0.9 μM each diagnostic primer, 0.25 μM diagnostic probe, and 1x final of TaqMan Universal PCR Mastermix (Applied Biosystems) in a reaction volume of 25 μl. The TaqMan PCR conditions used were as described in TaqMan guidelines. Each sample was analyzed in triplicate. As negative controls, we used both no-template and DNase-treated non-reverse-transcribed mRNA samples; no significant amplification was observed in these samples. *rp49* transcript levels were used as an endogenous normalization control for RNA samples, and relative mRNA abundance was calculated using the comparative delta-Ct method. Reference mRNA is noted in figure legends. Quantitative RT-PCR analysis was performed on at least two independent RNA preparations, with similar results.

## Abbreviations

AL: antennal lobe
*Bx*: *Beadex*
cDNA: complementary DNA
DA: dopamine
DAT: dopamine transporter
GFP: green fluorescent protein
*hdp*: *heldup*
*hid*: *head involution defective*
l-LN_v_: large ventral lateral neuron
LD: light–dark
LIM-HD: LIM-homeodomain
LMO: LIM-only
LN_v_: ventral lateral neuron
MB: mushroom body
*pdrm*: *pipedream*
s-LN_v_: small ventral lateral neuron
SCN: suprachiasmatic nucleus
*TeTx*: tetanus toxin light chain
*TeTx^in^*: inactive tetanus toxin light chain
VNC: ventral nerve cord
